# Evolution-guided phage therapy for antimicrobial-resistant *Acinetobacter baumannii*: from resistance evolution and genomic plasticity to receptor-associated fitness trade-offs and clinical translation

**DOI:** 10.3389/fmicb.2026.1910892

**Published:** 2026-10-19

**Authors:** Chuhan Wang, Guoliang Zhang, Honglan Huang

**Affiliations:** 1 Department of Pathogen Biology, College of Basic Medical Sciences, Jilin University, Changchun, Jilin, China; 2 Affiliated Hospital of Inner Mongolia University for Nationalities, Tongliao, Inner Mongolia, China

**Keywords:** *Acinetobacter baumannii*, evolutionary steering, multimodal antimicrobial therapy, phage therapy, precision phage therapy

## Abstract

Carbapenem-resistant *Acinetobacter baumannii* (CRAB) is sustained by environmental persistence, genomic plasticity, biofilm-associated tolerance, and rapid adaptation under antibiotic pressure. These features constrain conventional antibiotic regimens and make CRAB a relevant test case for evolution-guided antimicrobial control. This review first synthesizes how resistance architecture and genomic plasticity sustain multidrug resistance and clinical persistence. It then evaluates bacteriophages as selective agents that can alter the adaptive routes available to bacterial populations. We distinguish direct phage-mediated killing from evolutionary steering, in which receptor-dependent escape may create collateral costs in capsule or envelope function, biofilm formation, virulence, immune evasion, or antibiotic susceptibility. Receptor-associated claims are organized into three evidence categories: direct matched phage-escape evidence, indirect receptor/function evidence, and cross-species hypotheses. Direct matched evidence is concentrated in defined capsule-associated systems and supports context-dependent trade-offs, not a universal class effect. Animal studies support activity in specific systems, whereas a case-level clinical evidence table shows heterogeneous formulations, routes, co-interventions, and escape measurements; these reports primarily establish feasibility and treatment-associated observations, not general efficacy or clinical steering. Delivery and engineering strategies, rapid molecular profiling, and mechanistic models are therefore assessed as indirect evidence or testable design hypotheses rather than as equivalent support for steering. Translation also requires product quality, co-localized exposure, and serial measurement of burden, escape, phenotype, and coverage. The central challenge is to identify, exploit, and monitor a measurable and potentially transient window of collateral vulnerability rather than assume a stable evolutionary trap.

## 1 Introduction

The global rise of antimicrobial resistance (AMR) has increased the clinical priority and treatment burden of *Acinetobacter baumannii*, a hospital-associated opportunistic pathogen that is increasingly difficult to contain with conventional pharmacotherapy (Aggarwal et al., 2024; Mestrovic et al., 2025). In intensive care units (ICUs) worldwide, carbapenem-resistant *A. baumannii* (CRAB) is frequently associated with limited treatment options and poor outcomes in ventilator-associated pneumonia (VAP), persistent bloodstream infections, and complex soft-tissue infections (Wang et al., 2024).

*Acinetobacter baumannii* also shows pronounced environmental hardiness, persisting on abiotic surfaces for extended periods and tolerating desiccation (Lucidi et al., 2025). This physical persistence is coupled to genomic plasticity that enables rapid reconfiguration of resistance determinants under stress (Lucidi et al., 2024). Together, persistence and genomic plasticity allow resistance determinants and physiological states to persist or change during treatment.

Conventional antibiotic regimens are selected primarily for activity, exposure, and infection-site suitability, rather than for a prespecified bacterial evolutionary response. Effective killing can nevertheless enrich resistant variants when exposure removes susceptible subpopulations (Mehrotra et al., 2023). Separately, horizontal gene transfer provides additional routes for acquiring and disseminating resistance determinants: *A. baumannii* is naturally transformable, and conjugative systems provide another route for mobile-DNA transfer (Vesel et al., 2023; Zhang et al., 2025). Biofilms common in VAP and catheter-associated infections provide dense, spatially structured populations and extracellular DNA, although transfer of any resistance determinant remains conditional on the element, recipient, and local environment. The biofilm matrix also creates diffusion gradients and physiological refuges that expose some cells to sub-inhibitory antibiotic concentrations or permit slow-growing and persister cells to survive. This phenotypic tolerance is not itself heritable resistance, but recurrent survival and regrowth create repeated opportunities for selection and mutational diversification (Khalil et al., 2021; Yu et al., 2024; Olea-Ozuna et al., 2025).

Therapeutic failure reflects interacting genetic resistance, lineage structure, physiological tolerance, and infection-site heterogeneity rather than one isolated mechanism. Global and lineage-specific genomic analyses show that carbapenem-resistant *Acinetobacter baumannii* evolves within diverse clonal backgrounds and resistance-gene contexts (Müller et al., 2023; Abu Sabah et al., 2025). Current therapeutic reviews also document persistent limitations of available and emerging antibacterial options (Macesic et al., 2025; Dubey et al., 2025). These observations expose the conceptual gap addressed here: bacterial-burden reduction, resistance suppression, mechanistic steering, and clinical benefit are distinct outcomes and cannot be used as substitutes for one another. For prospective experimental and clinical-trial design, this systems-level framework comprises four core operations: representing the baseline population, applying a selective pressure with a defined escape hypothesis, measuring survivor phenotype and target-site exposure, and evaluating prespecified adaptation or stopping criteria when the predicted constraint is absent or lost. Importantly, these represent candidate validation principles rather than clinical recommendations for bedside patient management. Table 1 compares these therapeutic objectives and their evidentiary endpoints without presuming antibiotics to be intrinsically evolution-uninformed or phage regimens to be intrinsically evolution-informed. Sections 2–6 subsequently develop these four operations, guided by the three-category evidence appraisal framework outlined below and systematically applied from Section 3.2 onward.

**Table 1:** Therapeutic objectives and evidentiary endpoints for antimicrobial strategies.

Therapeutic objective	Candidate strategy	Required evidentiary endpoint	Failure or non-equivalent observation	References
Bacterial killing	Any active antibiotic, phage, enzyme, or combination selected for the isolate and infected compartment	Prospectively evaluated strategy with qualified products, co-localized exposure, longitudinal sampling, and a matched non-steering comparator	Susceptibility, spot clearing, administered dose, or genome detection without productive activity and burden response	Zhang et al. (2024); Daubie et al. (2022); Tan et al. (2024)
Resistance suppression	Combination, cocktail, sequence, or source-control strategy intended to reduce expansion of resistant subpopulations	Resistant-fraction or genotype-frequency trajectory plus killing/regrowth under matched exposure	Short-term burden reduction without measuring resistant fractions; resistance may still emerge	Wang et al. (2021); Kim et al. (2024); Mukhopadhyay et al. (2023)
Mechanistic evolutionary steering	A selective pressure paired with a prespecified escape state and survivor-tested complementary pressure	Predicted-state enrichment and a quantitative collateral phenotype in the same recovered population	Phage resistance, receptor association, altered expression, or collateral activity measured in an unrelated population	Gordillo Altamirano et al. (2021); Luo et al. (2026); Liu et al. (2025)
Clinical benefit from steering	Prospectively evaluated strategy with qualified products, co-localized exposure, longitudinal sampling, and a matched non-steering comparator	Persistent actionable phenotype, target-site activity, and improvement in a clinically relevant comparative outcome	Feasibility, uncontrolled case improvement, or mechanistic steering without comparative benefit	Liu et al. (2025); ClinicalTrials.gov, 2025

### 1.1 Literature search and evidence appraisal

This narrative review focuses on CRAB as the primary clinical problem and follows a structured narrative search rather than the prespecified protocol of a systematic review or meta-analysis. Because the original search log was not retained, the literature search was reconstructed during revision to make source selection auditable. PubMed/MEDLINE and Europe PMC were searched from database inception through September 1, 2026. The common search combined (*Acinetobacter baumannii* OR carbapenem-resistant *Acinetobacter baumannii* OR CRAB) with (phage* OR bacteriophage*). Four structured concept blocks then added terms for (i) receptors and adsorption (receptor*, adsorption, capsule, capsular, outer membrane, lipooligosaccharide, LOS); (ii) phage resistance or escape and its consequences (resistan*, escape, fitness, virulen*, collateral sensitivity, resensiti*, trade-off, tradeoff); (iii) phage-antibiotic combinations (antibiotic*, combination, synerg*, sequential); and (iv) clinical translation (clinical, patient*, compassionate, trial, pneumonia, bacteremia). For each database, the common block was executed alone as a baseline search and combined separately with each of the four concept blocks, yielding five queries per database (ten queries in total). Crossref records were consulted where DOI or publication metadata required verification, and ClinicalTrials.gov, FDA, EMA, and EDQM/European Pharmacopoeia records were checked for trial and regulatory status. Relevant studies not captured by this structured narrative search may reflect the scope of the queries rather than true absence from the literature.

Primary *A. baumannii* studies were eligible when they reported phage susceptibility or productive infection, receptor or adsorption experiments, recovered escape variants, matched collateral phenotypes, phage-antibiotic combinations, infection models, or clinical treatment observations. Three evidence categories organize the review, with claim-specific classification: one study may directly support an escape phenotype while providing only candidate evidence for the receptor that produced it. Clinical cases and expanded-access reports were used for feasibility, safety, or treatment-associated observations, not as controlled efficacy or clinical steering evidence; official trial, regulatory, and pharmacopoeial records were used for current-status claims. Sources without a direct relationship to *A. baumannii* biology, phage mechanisms, resistance evolution, product quality, safety, efficacy, or a relevant translational constraint were excluded; studies in other organisms were retained only as explicitly labeled Category 3 mechanistic, design, counterexample, carrier, immune, or modeling evidence. Preprints could identify recent candidate studies but were labeled as non-peer-reviewed and were not used alone to establish receptor identity, a matched trade-off, clinical efficacy, or regulatory status; a peer-reviewed version was used when available. Records were screened by author-led appraisal against these principles.

Evidence classification followed explicit causal thresholds defined for this review. A receptor relationship was considered confirmed only when direct adsorption or binding evidence was linked to a defined bacterial determinant and supported by causal genetic perturbation, preferably with complementation or restoration. Association, altered expression, sequence prediction, spot clearing, host-range correlation, or loss of susceptibility alone was classified as candidate or indirect evidence. Direct evidence of a phage-resistance-associated trade-off required a phage-selected or independently recovered escape population linked to a defined genotype or heritable state and a quantitative collateral phenotype measured against the parental population under matched conditions. Growth effects, independent receptor-function studies, omics associations, docking, or cross-species analogy alone did not meet this threshold, and clinical exploitability additionally required longitudinal enrichment of the predicted escape state, persistence of the collateral phenotype, co-localized complementary activity, and a better relevant outcome than matched non-steering care.

## 2 Adaptive resistance networks and evolutionary plasticity

### 2.1 Networked resistance architecture

The resilience of *Acinetobacter baumannii* to chemotherapy arises from interactions among genetic determinants, envelope physiology, regulatory coupling, and community-level protection (Sharma and Lakhanpal, 2025; Iovleva et al., 2022), so the effect of any one resistance mechanism depends on the state of the others. Permeability barriers reduce influx (Daruka et al., 2025), efflux lowers intracellular exposure (Maharramov et al., 2025), and biofilm-associated protection reshapes drug access and growth physiology (Darby et al., 2024); compensation after single-node intervention is a testable possibility, not an assumed outcome.

At the envelope level, reduced permeability, RND-family efflux, and beta-lactamase activity contribute jointly but do not form a universal ordered barrier. Among 90 clinical isolates, MIC associations varied by drug and lineage: OXA-24 or OXA-58 with carbapenem resistance, AdeABC or AdeIJK overexpression mainly with aminoglycoside, tigecycline, and minocycline phenotypes (Rumbo et al., 2013); *ISAba10* could both increase adjacent *blaOXA-23* expression and disrupt *carO*, yet an isolate retaining intact *carO* had similar MICs, so porin loss cannot be assigned a fixed contribution from genotype alone (Lee et al., 2011), a contextual dependency further compounded by biofilm-associated spatial heterogeneity (Section 2.3).

Collateral cross-resistance makes this interconnectivity consequential: upregulated broad-substrate efflux remodels susceptibility across quinolones, tetracyclines, and other classes (Salehi et al., 2021; Zahedani et al., 2021), turning exposure history into a predictor of multi-class non-susceptibility while single-node perturbation is offset by coupling and compensation (Kang and Boll, 2022). Strategies must therefore target multiple coupled nodes or exploit constraints the network cannot satisfy simultaneously.

Regulatory state alters this network without changing gene content: *aamA* deletion in strain ATCC 17978 changed genome-wide transcription and reduced efflux-associated resistance while altering vesicle production, biofilm, and virulence (Yang et al., 2023), and A118 methylation signatures gated acceptance of transforming DNA (Vesel et al., 2023). Both control points are strain- and condition-dependent, so methylation status alone cannot infer a clinical resistance phenotype.

### 2.2 Genetic mobility and clonal dissemination

Genomic plasticity supports long-term adaptation by allowing *A. baumannii* to acquire, recombine, and disseminate resistance determinants through mobile genetic elements (MGEs) (Naseef Pathoor et al., 2025). The clinically relevant unit is therefore often a population whose resistance repertoire can change over epidemiologically short timescales (Scribner et al., 2020; Liu C. et al., 2022; Luo Q. et al., 2024).

A central structural basis is the Acinetobacter resistance genomic island (*AbaR*), a mosaic platform whose content and insertion site vary among lineages (Bi et al., 2019). Class 1 integrons reorder resistance-gene cassettes but move mainly with their embedding transposons, plasmids, or islands, while insertion sequences and composite transposons mobilize linked loci, interrupt host genes, and supply outward-facing promoters. *ISAba1* upstream of *blaOXA-23*-like or *blaOXA-51*-like loci increases carbapenemase expression, yet insertion position alone does not predict resistance (Palmieri et al., 2020; Gautam et al., 2023; Carascal et al., 2024; Hu et al., 2024), and long-read analyses place these elements with resistance genes on both chromosomes and plasmids (Cerezales et al., 2020).

Plasmids add a transferable layer of dosage and regulation: conjugation moves self-transmissible plasmids, transformation imports extracellular DNA, and methylation-sensitive restriction favors compatible donors (Vesel et al., 2023; Zhang et al., 2025). An element persists only if replication, partitioning, and selection offset its burden, and low costs, co-selection, or improved persistence stabilize costly elements that successful clones then disseminate (Novović and Jovčić, 2023; Ridenhour et al., 2017; Metzger et al., 2022). Gene presence is an incomplete predictor: copy number, insertion site, promoter orientation, cassette order, integrity, and clonal background all influence phenotype.

Genetic mobility changes the unit of control to a linked module in a transmissible clone: because one antibiotic can co-select other genes on the same element, curtailing the selective advantage of such a module depends on adequate source control, verified target-site exposure, limiting the window of selection, and applying a complementary pressure with demonstrated activity against surviving subpopulations. *AbaR* profiles are lineage-associated (Bi et al., 2019) and related contexts span chromosomes and plasmids (Cerezales et al., 2020); stewardship, exposure control, and infection prevention remain the defensible constraints as conjugation suppression stays experimental.

### 2.3 Biofilm as an ecological resistance strategy

Clinical persistence is tightly linked to the biofilm-associated state. The polysaccharide, protein, and DNA matrix modifies transport and retains cells, while nutrient and antimicrobial gradients create zones with different exposures: peripheral cells meet lethal concentrations, and deeper regions contain slow-growing or persister cells under subinhibitory exposure. In an ST1894 model, sub-MIC imipenem or colistin altered matrix, quorum-sensing, efflux, and protein-synthesis transcription consistent with increased biofilm and reduced metabolism (Shenkutie et al., 2022). Such survival is phenotypic tolerance, not heritable resistance; recurrent survivors repopulate the infection and repeatedly expose a heterogeneous population to selection. Regulation is context-dependent: *AbaR* contributed to biofilm formation in the tested system (Sun and Xiang, 2021).

Biofilms also change inheritance of resistance: cell–cell contact and extracellular DNA enable conjugation and transformation, and experimental evolution showed that biofilm growth preserves multidrug-resistance plasmids without antibiotic selection (Ridenhour et al., 2017; Metzger et al., 2022). Spatial refuges maintain susceptible, tolerant, and resistant subpopulations simultaneously, allowing relapse after an apparently effective planktonic result. Experimental evaluation must therefore measure killing and regrowth within a representative model, phenotype recovered survivors, and establish whether reduced susceptibility persists following biofilm dispersal. Because matrix disruption alone is insufficient, serial sampling must distinguish the enrichment of a resistant lineage from a reversible tolerant reservoir-a requirement that motivates the targeted strategies developed in Sections 3.3 and 4.2.

### 2.4 Evolutionary cost and fitness trade-offs

Resistance determinants, envelope remodeling, and efflux systems frequently impose context-dependent growth, competitive, or virulence costs outside the selecting pressure (Yoon et al., 2016; Zhao et al., 2022; Tagueha et al., 2025). Such limits on simultaneous physiological optimization provide the conceptual foundation for evolutionary steering, wherein survival under one selective force impairs performance under another.

For instance, envelope remodeling that confers resistance to last-line agents can concurrently introduce membrane fragility (Zhao et al., 2022). Similarly, direct isogenic competition demonstrated that overexpression of AdeABC, AdeFGH, or AdeIJK reduced *in vitro* competitive fitness, although *in vivo* outcomes varied by pump system and infection route (Yoon et al., 2016)—highlighting that fitness costs negligible in nutrient-rich broth can become pronounced under nutrient-limited or competitive host conditions (Morris et al., 2024).

Collateral sensitivity—defined as heightened susceptibility to a secondary pressure following adaptation to a primary agent—fundamentally requires demonstration in paired ancestral and adapted populations. Defined *A. baumannii* phage-escape systems fulfill this criterion by recovering capsule-loss or *gtr*9 variants with verified re-sensitization to complement or beta-lactams (Gordillo Altamirano et al., 2021; Luo et al., 2026). Rigorous steering frameworks therefore depend on paired ancestral-versus-escape evaluations that quantify productive infection, escape frequency, matched collateral phenotypes, and their stability under targeted exposure (Oyejobi et al., 2024; Manley et al., 2024).

Given that branched resistance networks facilitate alternative evolutionary bypasses, candidate selection must evaluate receptor usage, escape frequency, and clonal diversity rather than lytic clearing alone, ensuring reproducible vulnerabilities across distinct backgrounds. These steering constraints and clinical-benefit criteria are systematically formalized in Section 3.2, and subsequently operationalized for schedule hypotheses and prospective validation in Sections 4.1 and 5.3.

## 3 Mechanistic reconstruction of phage therapy

### 3.1 Molecular recognition and the lytic cycle

Phage susceptibility begins when a virion receptor-binding protein, usually tail-fiber or tailspike-displayed, contacts an accessible surface ligand; in *Acinetobacter baumannii* these include K-locus capsular polysaccharides, outer-membrane proteins, and LOS glycans. Capsule may mask a deeper receptor, and tailspikes commonly carry depolymerase activity. StAb1 needed a specific capsule, StAb2 used capsule-occluded porin CarO in acapsular cells, and StAb3 needed a broad glycan receptor (McCalla et al., 2025); species or K-locus prediction shortlists a phage without establishing accessibility, host range, or productive infection.

After adsorption, productive infection requires genome entry, defense survival, replication, assembly, and lysis; receptor binding or spot clearing alone is insufficient, and biofilm matrices can physically mask receptors or prevent viral access. Local amplification depends on host density, receptor masking, exposure, and clearance (Chae, 2023; Cha et al., 2025).

Molecular recognition depends on population state: AbaM alters quorum sensing, pili, biofilm, and virulence but requires a defined adsorption test (López-Martín et al., 2021); BfmRS modulation changed Loki adsorption, replication, and killing (Bai et al., 2023); predicted restriction-modification, CRISPR-Cas, and abortive-infection loci do not establish activity against a therapeutic phage (Ambroa et al., 2022); horizontal gene transfer alters efficacy only when acquired DNA changes a receptor, its regulation, or a post-adsorption defense. Mechanistic attribution should combine escape clones, sequencing, perturbation and complementation, assays, and time-resolved adsorption, with plating efficiency, growth, progeny, and killing/regrowth distinguishing binding from productive infection across diverse clinical isolates (McCalla et al., 2025), providing the causal baseline for evaluating evolutionary steering (Section 3.2).

### 3.2 Phage-induced evolutionary steering

Phage treatment may kill susceptible bacteria, suppress resistance by broadening coverage, or steer evolution by enriching a heritable state with a measurable collateral phenotype; steering is probabilistic, the selected state may be favorable, neutral, or adverse, and burden reduction alone demonstrates killing rather than steering (Figure 1). Three explicit evidence categories (Subsections 3.2.1–3.2.3) govern the mechanistic and translational claims evaluated across this review. Classification is assigned to each claim, not automatically to an entire study, because a study may directly demonstrate a matched escape phenotype while leaving the responsible receptor incompletely resolved.

**Figure 1:**
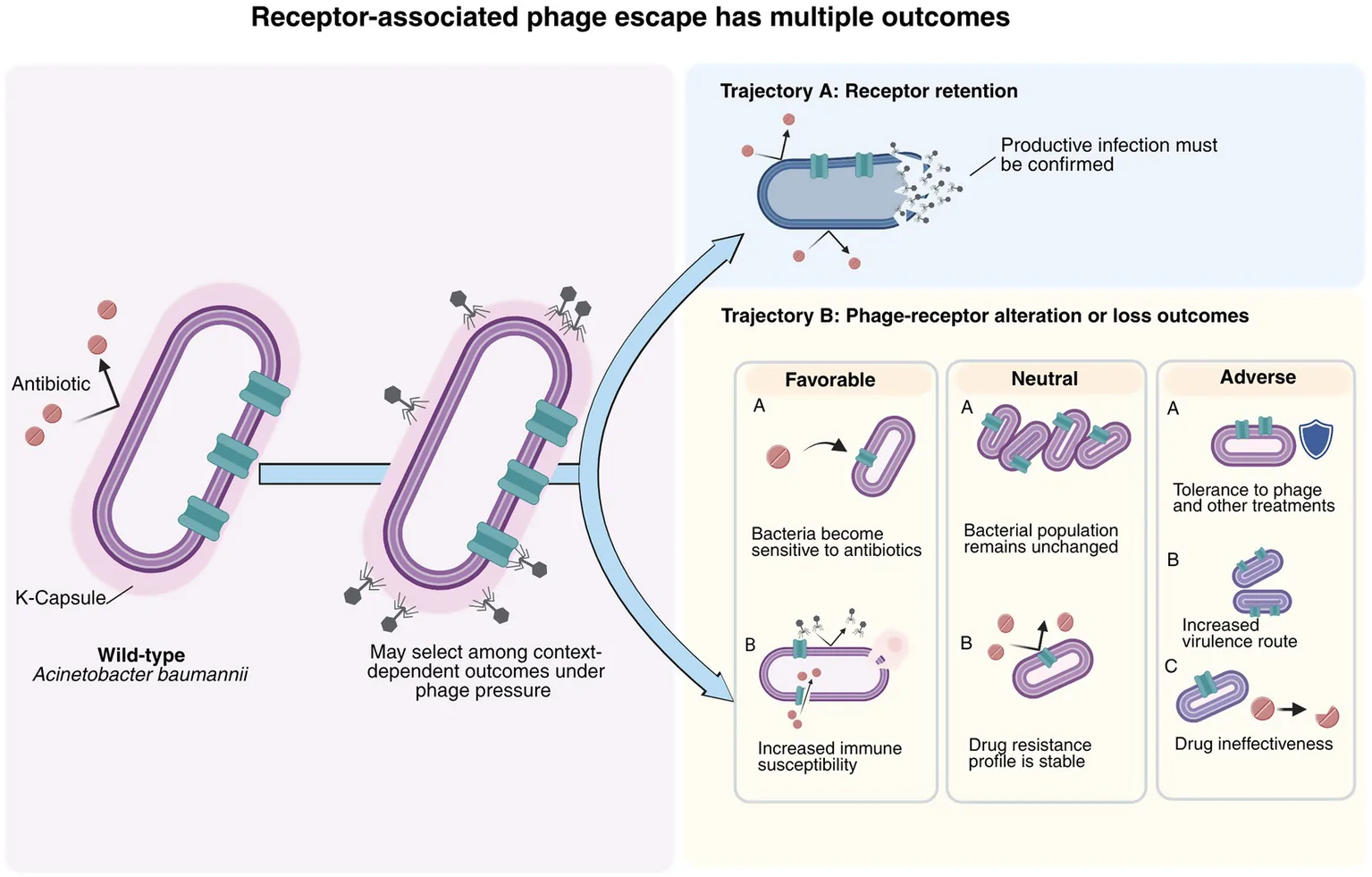
Context-dependent outcomes of receptor-associated phage escape in *Acinetobacter baumannii*. The schematic separates killing without demonstrated steering (receptor retention) from three possible escape outcomes. A favorable branch requires a measured vulnerability in the recovered population, such as reduced virulence, greater immune susceptibility, or antibiotic re-sensitization. A neutral branch retains fitness without an actionable vulnerability. An adverse branch includes cross-resistance, compensatory recovery, increased resistance to a companion agent, or alternative high-fitness escape. Receptor alteration alone does not determine the branch; genotype, strain, compartment, exposure, and time must be measured. The illustrated surface structure is not a universal receptor.

The central framework uses four prespecified conditions. Predicted-state enrichment means that phage exposure reproducibly increases a defined heritable escape state relative to baseline or a matched control; burden reduction without enrichment is killing, not steering. A matched collateral phenotype is a quantitative vulnerability measured in that same recovered population against its parental comparator; phage resistance or an independent receptor-function study alone is insufficient. Co-localized complementary activity requires the second pressure to remain active against the selected population where and when infective phage and bacteria overlap. Persistence requires the genotype–phenotype link and actionable vulnerability to endure over the prespecified observation or treatment window rather than disappear through regulation, alternative escape, or compensation. The first two conditions support a mechanistic steering claim. Clinical benefit additionally requires the latter two and a better relevant outcome than matched non-steering care. These operational criteria serve as the reference benchmark applied across subsequent sections, within which clinical steering remains unproven.

#### 3.2.1 Category 1: direct matched *Acinetobacter baumannii* phage-escape evidence

Direct matched evidence requires phage-selected or independently recovered *A. baumannii* escape populations, a linked genotype or heritable state, and a quantitative collateral phenotype measured against the parental population under matched conditions. The strongest examples are capsule-associated. Capsule-deficient mutants with biosynthesis-gene lesions showed disrupted adsorption with increased complement or beta-lactam susceptibility and reduced fitness (Gordillo Altamirano et al., 2021); a *gtr*9 stop-gain variant linked resistance and reduced capsule to impaired biofilm, zebrafish virulence loss, carbapenem re-sensitization, and pB23-meropenem activity (Luo J. et al., 2024; Luo et al., 2026); and in colistin-resistant backgrounds, *lpxC/lpxD* disruption constrained routes while compensatory changes expanded them (Hao et al., 2026). This is matched, background-specific evidence, not a general LOS map or envelope effect.

This matched tier also captures unfavorable or neutral outcomes: Manley et al. linked resistance to a putative insertion and reduced larval virulence but observed regrowth, cross-resistance, and weak rescue (Manley et al., 2024), and an early growth deficit in ATCC 17978 decontamination derivatives was not significant at 24 h, establishing no compensation or clinical window (Chen et al., 2025). Durability requires serial confirmation that genotype and phenotype persist under an active complementary pressure; recovery, alternative escape, cross-resistance, or compensation closes it.

Direct matched evidence remains concentrated in a few capsule-associated systems; generality requires testing across receptors, lesions, backgrounds, infection states, and companion pressures — one model is a candidate rule, not a clinical generalization.

#### 3.2.2 Category 2: indirect *Acinetobacter baumannii* receptor/function evidence

Indirect organism-specific evidence encompasses candidate receptors, adsorption determinants, or functional phenotypes that lack the complete matched escape chain. Phage-tolerant isolates showed reduced adsorption, altered antibiotic susceptibility, and downregulation of *ompA* and other OMPs, but expression does not establish OmpA as the causal receptor or make re-sensitization generalizable (Nayak et al., 2025). Independent OmpA, LOS, efflux, and capsule studies identify testable consequences, not matched steering (Beceiro et al., 2014; Yoon et al., 2016; Akoolo et al., 2022).

Receptor assignment remains claim-specific: capsule may be a receptor or mask another determinant, and some phages use sequential routes (Bai et al., 2023; McCalla et al., 2025); a recurrent *gtr*76 deletion in serial isolates accompanied phage insensitivity but not a matched collateral phenotype or steering (Liu M. et al., 2022), and reduced adsorption leaves a candidate until causal perturbation and preferably complementation. Complete LOS loss can confer colistin resistance, so envelope disruption cannot imply a favorable polymyxin trade-off.

#### 3.2.3 Category 3: cross-species mechanistic hypotheses

Cross-species mechanistic hypotheses can guide experimental design but cannot populate an *A. baumannii* receptor map. In *Pseudomonas aeruginosa*, OprM- or O-antigen-associated escape increased selected susceptibilities (Chan et al., 2016; Nakamura et al., 2021), whereas *Escherichia coli* TolC mutations often produced resistance without altered susceptibility (Tamer et al., 2021). These are design and counterexample evidence, not proof that an Ade component is an *A. baumannii* receptor (Fujiki et al., 2025). Cross-species immune, carrier, engineering, CRISPR, and modeling studies follow the same hypothesis-only basis.

Table 2 applies these categories to receptor-associated claims. Category 2 or 3 evidence can justify an experiment but cannot be promoted to Category 1 by association, sequence prediction, altered expression, spot clearing, docking, omics, or modeling.

**Table 2:** Receptor-confirmation status and evidence limits for *Acinetobacter baumannii* steering claims.

Evidence category	Structure	Receptor status in *A. baumannii*	Confirmation basis	Collateral/escape evidence	Maximum defensible inference	References
Category 1 in selected capsule systems; otherwise claim-specific	K-capsule and capsule biosynthesis/export loci	Confirmed for defined phage-capsule relationships; not a universal receptor class	Direct adsorption/productive-infection dependence linked to defined capsule determinants, with causal perturbation and restoration/complementation in selected systems.	Matched escape populations link specific capsule lesions to complement, virulence, biofilm, or antibiotic phenotypes	The cited phage-strain-lesion relationship only; capsule loss may also be neutral, adverse, or bypassed	Bai et al. (2023), McCalla et al. (2025), Gordillo Altamirano et al. (2021), Luo et al. (2026), and Liu et al. (2025)
Category 2	OmpA and other non-efflux outer-membrane proteins	Candidate; OmpA is not confirmed as the causal receptor in the cited phage-tolerant system	Reduced adsorption plus altered OMP expression; no complete causal perturbation-and-complementation receptor chain.	Altered antibiotic susceptibility/function is organism-specific but not a reproducible matched steering rule	The cited evidence supports prioritizing receptor testing; OmpA-mediated re-sensitization is not established	Nayak et al. (2025), Liu et al. (2021), and Viale and Evans (2020)
Category 1/2, claim-specific	LOS outer-core glycans and lipid A-associated structures	A defined outer-core determinant is confirmed as a secondary infection factor for AB_SZL3/AB_SZL4	*gtr*OC3 perturbation/complementation altered productive infection while initial adsorption persisted; other LOS/lipid A claims are functional or associative.	Clinical/selected isolates show heterogeneous OC/K-locus escape and collateral phenotypes; complete LOS loss can increase colistin resistance	Distinguish primary receptor, secondary infection determinant, and independent envelope function; the cited data do not support inferring a polymyxin benefit from LOS disruption	Liu et al. (2025) and Beceiro et al. (2014)
Category 3	Ade/efflux-associated outer-membrane components	Unconfirmed in *A. baumannii* therapeutic phage systems	*A. baumannii* efflux-function studies lack a demonstrated phage receptor chain; OprM evidence is from *P. aeruginosa*	Cross-species favorable trade-off and TolC counterexample show that efflux association does not guarantee re-sensitization.	Experimental hypothesis only until receptor use, pump impairment, and matched phenotype are co-demonstrated in *A. baumannii*	Chan et al. (2016), Tamer et al. (2021), Yoon et al. (2016), and Sharma et al. (2023)

### 3.3 Biofilm disruption mechanisms

Within this evidence framework, organism-specific biofilm-component and depolymerase studies represent indirect functional evidence (Category 2) unless paired with a matched phage-escape population, whereas staged carrier-mediated regimens remain cross-species design hypotheses (Category 3) until validated in CRAB. CRAB biofilm recalcitrance reflects a layered, strain-variable matrix: PNAG from the *pgaABCD* locus contributes substantially to volume and thickness (Choi et al., 2009), extracellular DNA is DNase-reducible (Sahu et al., 2012), and Csu pili mediate attachment while capsule and adhesins determine maturation and retention (Pakharukova et al., 2018); biomass removal without reaching slow-growing or resistant cells changes architecture without sterilization.

Matrix disruption must match the component present: capsule-specific tailspikes or depolymerases remove cognate polysaccharide and expose receptor-bearing or complement-sensitive cells but are capsule-restricted; for example, the phiAB6 tailspike reduced established biofilm and catheter colonization *in vitro* as a component-specific proof of principle (Shahed-Al-Mahmud et al., 2021). DNase is rational only where extracellular DNA is structurally important, proteolysis requires an accessible scaffold, and physical source control remains distinct from and irreplaceable by biomass reduction.

Capsule or matrix disruption increases access substrate-, strain-, and agent-specifically: the Dpo71 depolymerase stripped capsule, increased colistin binding, disrupted biofilm, and enhanced colistin activity (Chen et al., 2022). Because matrix opening can release viable cells, dispersal strategies require an active killing pressure, evaluated by biomass, viable burden, penetration, phage titer, regrowth, and survivor susceptibility rather than crystal-violet loss alone. Figure 2 consequently organizes matrix opening, phage release and lysis, and antibiotic exposure as a hypothetical, testable sequence rather than a guaranteed eradication pathway.

**Figure 2:**
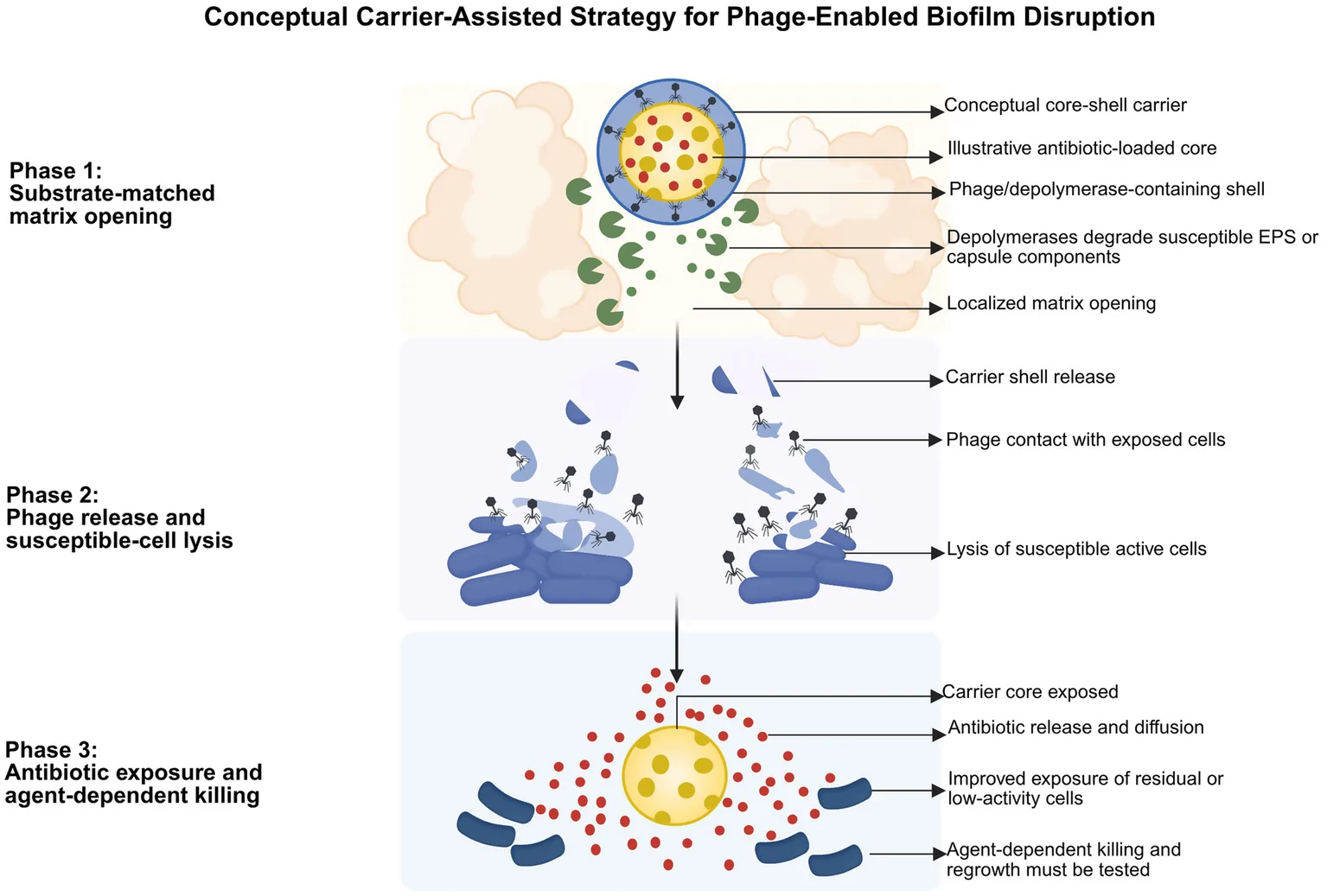
Hypothetical design framework for carrier-assisted phage-enabled biofilm disruption. The entire sequence is hypothetical for CRAB. Phase 1 tests substrate-matched matrix opening by a demonstrated depolymerase; Phase 2 requires productive phage infection and viable-burden/regrowth measurement; Phase 3 tests whether a companion agent reaches and acts on the residual population. *A. baumannii*-specific matrix and depolymerase findings motivate Phases 1–2, whereas integrated staged carrier evidence is largely cross-species or platform-level. Improved access is not eradication, and each arrow is a validation dependency rather than an established clinical sequence.

### 3.4 Phage-host-immune interactions

Evidence linking phage selection to host immunity spans three distinct tiers: increased complement susceptibility in matched capsule-deficient *A. baumannii* variants represents direct matched escape evidence (Category 1); independent clearance and cytokine responses provide indirect, organism-specific observations (Category 2); and immunophage cooperation demonstrated in other pathogens serves strictly as a cross-species mechanistic hypothesis (Category 3). Systemic phages meet two immune processes with opposite consequences: phagocytes sequester circulating particles, and repeated exposure generates neutralizing antibodies; indeed, cross-reactive neutralizing antibodies contributed to a non-Acinetobacter compassionate-use failure, emphasizing that binding and functional neutralization must be distinguished (Tan et al., 2024; Gordillo Altamirano et al., 2026). Antibacterial effectors complete bacterial control after lysis lowers burden, but paradigms established in immunocompetent Pseudomonas models or specific capsule variants cannot be extrapolated to all infection routes or immunocompromised hosts (Roach et al., 2017; Weissfuss et al., 2025; Gordillo Altamirano et al., 2021).

Inflammatory risk has two sources. Product-borne pyrogen persists unless manufacturing removes it, whereas treatment-emergent LOS is released from bacteria killed at the infection site; its impact depends on burden, compartment, killing rate, co-medication, and clearance, so phage dose alone is an insufficient surrogate. In an *in vitro Escherichia coli* comparison, phages released less free endotoxin than tested beta-lactams, demonstrating that rapid lysis does not inherently maximize endotoxin exposure; however, this finding cannot be generalized to *A. baumannii* infection or septic patients (Dufour et al., 2017). Studies should pair burden and phage kinetics with endotoxin measurement, inflammatory markers, hemodynamics, and organ-injury endpoints; route, infusion rate, and escalation are monitored controls, but no validated lysis threshold predicts a cytokine or clinical event.

### 3.5 Preclinical and clinical evidence

*In vitro* assays establish adsorption, infection, killing, regrowth, and escape under defined conditions, not exposure, immune effects, safety, or efficacy (Daubie et al., 2022); *in silico* models depend on structure, identifiability, calibration, and external validation, and an integrated pneumonia model illustrates the method without *A. baumannii* doses (Delattre et al., 2022); animal studies add distribution, clearance, immunity, and safety observations for a defined system (Choi et al., 2025); human reports establish feasibility. No completed controlled study has established *A. baumannii*-specific efficacy. A registered randomized trial of nebulized cocktails for Gram-negative ventilator-associated pneumonia was not yet recruiting when rechecked on August 3, 2026. Any eventual organism-specific inference will require prespecified, adequately represented species analyses (ClinicalTrials.gov, 2025). Translation requires concordance across tiers.

#### 3.5.1 Preclinical animal model

Animal studies demonstrate activity in particular systems, not class effects: phages reduced burden or improved survival in defined *A. baumannii* mouse wound and systemic-infection models, with recent work linking efficacy to exposure or a selected second phage (Regeimbal et al., 2016; Gordillo Altamirano et al., 2021; Choi et al., 2025; Shao et al., 2026). These studies differ in strain, phage, inoculum, timing, route, anatomy, comparator, and immune context, so effect sizes should not be pooled without replication across representative isolates.

Preclinical programs should use panels spanning CRAB lineages, K and outer-core loci, resistance profiles, and infection sources; each phage-isolate pair requires productive-infection, killing/regrowth, and minority-escape testing. Mammalian studies need relevant antibiotic or immune contexts, replication, prespecified endpoints, randomization, blinding, and safety controls.

Longitudinal studies should measure burden, target-site phage, recurrence, toxicity, and multiple colonies, pairing sequencing and receptor tests with both susceptibilities, competition, biofilm, serum survival, and virulence. Translation requires reproducible activity, adequate exposure, and a collateral phenotype persisting through the intervention window; short-term burden reduction does not establish durability.

#### 3.5.2 Clinical evidence: case reports and compassionate use

Published *A. baumannii* evidence remains dominated by compassionate use and small series. Table 3 separates six published patient-level records by infection, strain/K locus, phage and route, concurrent antibiotics or source control, resistance, escape genotype, collateral phenotype, outcome, and causal-inference strength. NR means that the primary report did not provide the field; it is not inferred from another patient, isolate, or experiment. The ratings distinguish evidence for a system-specific mechanistic chain from evidence for clinical benefit: even the strongest longitudinal escape record remains uncontrolled and cannot isolate phage effects from antibiotics, surgery, source control, coinfection, or immunity.

**Table 3:** Patient-level clinical evidence for *Acinetobacter baumannii* phage therapy and evolutionary steering.

Infection; strain/K locus	Phage(s); route	Concurrent antibiotics/source control	Phage resistance	Escape genotype	Measured collateral phenotype	Clinical outcome	Causal-inference strength	Source/case
Disseminated MDR infection with infected pancreatic pseudocyst; TP1-TP3; K locus NR	Personalized ΦPC/ΦIV, then ΦIVB; intracavitary plus intravenous	Multiple antibiotics, including meropenem/minocycline; percutaneous drainage	Yes; TP3 resisted both initial cocktails	NR	Altered capsule/antibiotic-response observations, but no unambiguous matched quantitative re-sensitization chain	Culture clearance, drains removed, discharged home, returned to work	Low for clinical attribution; low-to-moderate for mechanistic adaptation because serial resistance prompted reformulation, but antibiotics and drainage co-occurred	Schooley et al. (2017)
CRAB ventilator-associated pneumonia with *P. aeruginosa* coinfection; Ab059-R; K locus NR	AbW4878ø1; intravenous, then intravenous plus nebulized	Broad-spectrum antibiotics throughout; no separate source-control procedure reported	No; day-14 isolate remained susceptible	NR	NR	Two CRAB-negative sputum cultures, ventilator weaning, discharge to rehabilitation	Low; combined-regimen feasibility only	Rao et al. (2022)
XDR pulmonary infection with polymicrobial respiratory infection; sputum isolate; K locus NR	BA3; two inhaled 10-day courses	Continuous multidrug antibiotics; no additional source-control procedure reported	Suspected loss of activity during course 2; viable resistant isolate not demonstrated	NR	NR	First-course bacterial-DNA decline; later stable, ventilator weaning and rehabilitation transfer	Low; qPCR detected DNA, not a genotype-defined escape chain or phage-specific effect	Qu et al. (2024)
Recurrent XDR severe pneumonia with ARDS; sputum isolate; K locus NR	Patient-matched lytic phage related to phage Arbor; nebulized for 8 days	Intravenous polymyxin B, amikacin and fosfomycin; no source-control procedure reported	NR	NR	NR	Five consecutive negative sputum cultures with improved inflammatory, imaging and ventilation indices	Low; combined-regimen temporal association only	Lin et al. (2025)
Persistent polymicrobial aspiration pneumonia with KL2 CRAB; NAB01B and serial isolates	AB_CK1, AB_CK2, then AB_CK3; three nebulized courses	Isepamicin/other antibiotic courses and fluconazole for coinfection; source control NR	Yes; serial clinical resistant isolates	*gtr*OC3 OC-locus variants and *wzy*_KL2 stop-gain/truncation; progressive	Altered antibiotic MICs, biofilm, serum susceptibility and cell/larval virulence in serial clinical and/or representative resistant isolates	Cultures negative after course 3; no reported phage-related adverse event or recurrence over 28 months	Moderate for system-specific mechanistic signal; low for phage-specific clinical benefit because treatment was uncontrolled and polymicrobial	Liu et al., 2025, patient 1
*A. baumannii* pneumonia with co-pathogens; NAB09B/NAB10B; KL2	AB_CK2; nebulized twice daily for 21 days	Antibiotics tailored to co-pathogens; source control NR	No resistant isolate detected	No mutations reported between baseline/follow-up isolates	NR	Microbiological recovery and declining sputum Acinetobacter relative abundance; no adverse signal reported	Low; targeted-treatment feasibility only; patient 1 trade-off evidence cannot be transferred	Liu et al., 2025, patient 2

NR, not reported in the primary case report or its accessible tables/supplementary descriptions. Causal-inference strength concerns attribution of clinical benefit unless a mechanistic signal is explicitly specified; a single uncontrolled case cannot establish efficacy. “No source-control procedure reported” does not prove that none occurred outside the report. Escape genotype and collateral phenotype must come from the same patient’s recovered population and are not borrowed across cases.

The landmark case reported by Schooley et al. (2017) illustrates both the opportunity and the interpretive limits of adaptation. Serial resistance prompted reformulation of the ΦPC/ΦIV/ΦIVB phage regimen, but no linked escape genotype or unambiguous matched collateral phenotype established steering; recovery occurred during phage, antibiotics, and drainage.

The strongest clinical mechanistic signal was reported for Liu et al. (2025) patient 1: longitudinal KL2 isolates included phage-resistant OC/K-locus variants and progressive enrichment of a *wzy* mutant, with receptor perturbation/complementation and antibiotic, biofilm, serum, and virulence phenotyping across serial clinical and representative resistant isolates. Patient 2, however, had no recovered resistant isolate or matched collateral phenotype. Neither case establishes comparative efficacy because both received concurrent treatment and lacked a non-steering comparator.

Rao et al. (2022) reported improvement during a combined regimen using AbW4878ø1, and Lin et al. (2025) reported improvement during a combined regimen without a measured escape chain. Qu et al. (2024) observed an initial decline in sputum bacterial DNA followed by poor apparent activity during a second inhaled course, but did not recover and genotype a resistant isolate. These reports provide feasibility and monitoring contrasts, not effect estimates.

The microbiome observation in the Qu case does not establish a steering outcome: fecal qPCR detected phage DNA rather than infective phage, 16S data were relative, and critical illness plus concurrent antibiotics prevent attribution (Qu et al., 2024). It is retained only as an unresolved ecological signal, not as evidence of efficacy, safety, or an exploitable trade-off. Prospective microbiome assessment should distinguish direct productive infection of non-target bacteria from indirect shifts after target depletion or concomitant treatment by pairing phage DNA with recoverable infective phage and strain-resolved abundance with functional or metabolomic and mucosal or systemic immune endpoints.

Across Table 3, only one patient-level record approaches the matched mechanistic chain, and none establishes clinical benefit from steering. Prospective studies should therefore pair product-version traceability and co-intervention records with target-site infective phage, burden, regrowth, survivor genotype and phenotype, recurrence, toxicity, and comparison with a prespecified non-steering regimen.

## 4 Synergistic therapeutic architecture: multimodal integration

### 4.1 Phage-antibiotic evolutionary confinement strategy

This section applies the Section 3.2 framework to prospective schedule hypotheses that require validation in controlled studies; no controlled comparison of these schedules exists in *A. baumannii* infection. Simultaneous, sequential, replacement, and stopping decisions are therefore framed as prespecified trial variables for validation rather than as recommendations for current patient management. Selection-mediated synergy differs from two independent killing effects and cannot be inferred from a static FICI, which omits time-dependent replication (Orozco-Ochoa et al., 2025; Kim et al., 2024).

In the compassionate-use cases synthesized here, phages accompanied ongoing antibiotics (Schooley et al., 2017; Rao et al., 2022; Lin et al., 2025; Liu et al., 2025). This reviewed sample does not establish universal practice; because the courses were uncontrolled, they cannot separate any effect of schedule from antibiotics, surgery, source control, or host factors, and they motivate explicit tests of composition, timing, and exposure.

The receptor basis remains specific (Tarasenko et al., 2025). Across these routes, evidence strength varies with receptor biology: capsule-associated steering is supported by direct matched escape evidence (Category 1) only within defined phage-isolate pairs; LOS and lipid A modifications represent either matched steering or indirect candidate effects (Category 1/2) claim-specifically; OMP perturbations reflect indirect candidate observations (Category 2); and efflux components remain cross-species receptor hypotheses (Category 3). Capsule changes alter complement and surface protection; LOS or lipid A can impose costs or increase polymyxin resistance; OMP changes affect adhesion, biofilm, or permeability; efflux compromise requires demonstrated transport and susceptibility changes (Deng et al., 2025; Zhou et al., 2026). Nayak et al. linked phage-tolerant *ompA* downregulation to reduced adsorption and altered susceptibility, without making re-sensitization inevitable (Nayak et al., 2025).

One simultaneous-schedule hypothesis is that joint exposure improves population control when phage-susceptible cells predominate. It should be tested against the Section 3.2 criteria using measured survivor susceptibility rather than receptor-class assumptions; a simple probability argument is insufficient (Wang et al., 2018; Blasco et al., 2022). Co-administration can fail if antibiotics remove permissive hosts, receptor-negative cells expand, or compartments separate the pressures (Upmanyu et al., 2022; Sharma et al., 2023).

A prospective phage-first hypothesis is testable only when recovered survivors reproducibly acquire a vulnerability to the companion antibiotic before compensation; the *gtr*9 system and sequence-dependent results illustrate the rationale (Luo et al., 2026; Mukhopadhyay et al., 2026). Allocation or adaptation would follow a prespecified phenotype, not a fixed delay.

A sequential-schedule hypothesis would be rejected when phage exposure or amplification is inadequate, resistant cells lack the intended trade-off, the antibiotic is mismatched, or compensation closes the window (Choi et al., 2025). Receptor, susceptibility, killing/regrowth, and compartmental-exposure measurements should govern prespecified protocol adaptations; the evidence thresholds remain those in Section 3.2.

Prospective comparisons should estimate joint exposure-response across bacterial densities, phage inputs, attainable antibiotic exposures, and schedules while following viable bacteria, infective phage, regrowth, and resistant fractions (Orozco-Ochoa et al., 2025; Kim et al., 2024; Grygorcewicz et al., 2022; Mukhopadhyay et al., 2023). Kinetics, mechanism, titers, and clearance preclude a universal MOI, dose, interval, or order; redosing remains a protocol variable, with toxicity, neutralization, burden, microbiome, and dual-resistance constraints. Section 5.2 describes how these data could test, not determine, schedules.

### 4.2 Biofilm-directed delivery as control of spatial selection

The carrier architectures evaluated here represent cross-species design hypotheses (Category 3); although organism-specific matrix and depolymerase studies provide supportive functional context (Category 2), they do not yet demonstrate a coordinated, staged eradication strategy in CRAB. Nonuniform exposure can give antibiotics to some subpopulations and phage to others, enriching escape variants (Upmanyu et al., 2022; Sharma et al., 2023); delivery matters only when it improves spatial and temporal overlap of matched pressures.

A useful carrier must preserve infective titer, reach the infected compartment, release while susceptible bacteria remain for amplification, and avoid shielding the payload from receptor-bearing cells (Malik, 2021; Samananda Singh, 2024); co-delivered antibiotics must keep activity against the ancestral population or a measured escape phenotype.

Carrier choice follows the pressure mismatch, not material taxonomy: local retention for wounds and devices, aerosolization-compatible infectivity for pulmonary delivery, and measured target-site phage for systemic use. Because most carrier evidence comes from *Pseudomonas aeruginosa* or *Staphylococcus aureus*, each platform remains a CRAB hypothesis until it improves co-localized exposure versus matched free agents (Kim et al., 2021; Wroe et al., 2020; Cortés et al., 2024; Sawant et al., 2025).

Release timing must prevent single-pressure refuges: too-rapid or delayed release decouples phage amplification from companion activity (Temsaah et al., 2025). A staged design is relevant only if it opens a capsule- or matrix-rich compartment and then exposes survivors to an active companion pressure; the CRAB phage-Ce6 conjugate is a local proof of concept for separable receptor-dependent and photoactivated killing, not general evidence that carriers prevent resistance (Kebriaei et al., 2022; Erol et al., 2023; Su et al., 2025).

Preclinical qualification requires comparing free and formulated phage at equivalent starting infective titers; decisive evaluation endpoints include target-site infective recovery, receptor access, overlap with the companion pressure, viable burden, regrowth, resistant fractions, and collateral-phenotype persistence (Malik, 2021; Wroe et al., 2020; Temsaah et al., 2025; Sawant et al., 2025; Patpatia et al., 2022). Responsive or co-loaded systems must report cue specificity, false activation, response lag, and separate release profiles, since antibiotic class alters replication (Kim et al., 2024).

### 4.3 Programmable phages and CRISPR-based evolutionary control

Engineered-phage and phage-assisted CRISPR platforms remain cross-species design hypotheses (Category 3) until validated directly in *Acinetobacter baumannii*; most mechanistic foundations were established in *Escherichia coli*, *Pseudomonas aeruginosa*, or other model systems and cannot be assumed to operate equivalently in CRAB (Khambhati et al., 2023; Chaudhary et al., 2025). For CRAB, programmability is a design-and-validation problem grounded in capsule diversity, receptor heterogeneity, defense systems, and genomic safety.

Retargeting or broadening adsorption could increase coverage, and combining phages with nonredundant receptors could reduce escape from an entire cocktail (Jia et al., 2023; McCalla et al., 2025); the design must start from experimentally resolved capsule specificity (Drobiazko et al., 2022; Islam et al., 2024). Any engineered change requires testing for adsorption, infection, stability, host range, and escape genotype and phenotype; broader range is not favorable steering if resistance shifts to a high-fitness, high-virulence route.

Phage-assisted CRISPR delivery could remove resistance determinants or deplete a genotype, but present *A. baumannii* evidence does not establish a platform. Wu et al. (2022) characterized *pyrF*-based manipulation and type I-F CRISPR-Cas components, and Yu et al. (2024) showed that sub-MIC exposure altered I-Fb fitness costs; these findings reveal organism-specific physiological constraints rather than establishing efficient phage-mediated delivery in CRAB. Validation must measure delivery, cleavage, escape, transfer, and activity across clinical isolates.

A construct is useful only if it closes a defined escape route, redirects resistance toward a measured vulnerability, or enables rapid replacement; candidates should be compared with unmodified phages and receptor-diverse cocktails, with survivors genotyped and phenotyped. Engineering should make the adaptive landscape more measurable, not merely more complex.

Translational validation requires testing across capsule-diverse clinical isolates, with minimum evaluation gates including sequence stability, absence of lysogeny or cargo, host range, productive infection, killing and regrowth, escape genotype and phenotype, transfer, and model safety. Because phage-associated *blaOXA-23* spread illustrates dissemination risk, transfer should be measured under intended conditions (Abouelfetouh et al., 2022); until met, engineered breadth or cargo activity is an intermediate phenotype, not clinical benefit.

## 5 Decision architecture for *Acinetobacter baumannii* evolutionary steering

### 5.1 Rapid phenotyping for prospective protocol entry, omics for learning

A minimum candidate protocol-entry dataset would include a representative culture, productive-infection and killing/regrowth testing, susceptibility of baseline and recovered populations, and a qualified product definition (Humphries et al., 2021; Salam et al., 2023); these variables require validation as enrollment criteria, whereas standard antibiotic MIC determinations fail to capture phage adsorption, viral replication, or population regrowth.

Rapid species, capsule, K-locus, receptor, or defense genotyping may shortlist candidates, but receptor expression, physiology, and mixed subpopulations alter susceptibility: prediction narrows the phagogram without declaring productive infection.

Transcriptomics, proteomics, and metabolomics serve as indirect, organism-specific evidence (Category 2) within *Acinetobacter baumannii*, but revert to cross-species hypotheses (Category 3) when borrowed from other organisms; neither constitutes direct matched evidence (Category 1) unless paired with a verifiable phage-escape genotype and a quantitative collateral phenotype. Mechanistic studies should therefore compare matched baseline and escape populations under defined phage, antibiotic, and immune conditions, linking molecular changes to direct infection phenotypes; association alone does not establish causality, and no validated omics signature substitutes for direct phage-susceptibility testing for an individual CRAB isolate or clinical scenario.

The prospective tier uses phenotype and short-interval sampling; sequencing forms a parallel learning tier that updates receptor and bank annotations and model ranges. Multiomics remains a cross-case learning tool, not a protocol-entry gate.

### 5.2 Mechanistic models as scenario tests

Mechanistic mathematical models function as exploratory scenario tools (Category 3) unless calibrated and prospectively validated within the identical *Acinetobacter baumannii* strain-phage-companion-agent system; *in silico* curve fitting cannot generate direct matched steering evidence (Category 1). Populations should distinguish susceptible, infected, receptor-defined escape, and compensated states, and processes enter only when they alter a Section 3.2 criterion (Chae, 2023; Leung and Weitz, 2017). Cross-species models supply structures, not *Acinetobacter baumannii* parameter values.

Spatial structure is warranted only to capture plausible single-pressure refuges, and stochastic dynamics should be incorporated only when addressing extinction, alternative escape, cross-resistance, or compensation; joint models must couple state-dependent phage production to antibiotic effect (Summers and Kreft, 2022; Leclerc et al., 2022; Oromí-Bosch et al., 2023). Predictions should be challenged with withheld time courses, propagating uncertainty (Delattre et al., 2022); models may compare competing schedule hypotheses but cannot define a transferable clinical dose or override observed microbiological responses.

### 5.3 A two-tier prospective validation framework for *Acinetobacter baumannii* evolutionary steering

The upper tier is designed as a candidate experimental or clinical-trial pathway rather than a current-care algorithm. Protocol entry would document infection site and species, preserve diversity when feasible, and use capsule typing only to triage a panel; productive-infection, killing/regrowth, and compatibility tests remain necessary (Akoolo et al., 2022; Islam et al., 2024).

Serial samples measure burden, target-site phage, both susceptibilities, and survivor phenotype at prespecified points; pre-existing resistance can defeat a colony-derived match (Gordillo Altamirano et al., 2026), and substitution requires measured coverage loss and a qualified replacement.

K locus or algorithmic matching may rank candidates (Aggarwal et al., 2023; Liu et al., 2025); every prediction requires productive-infection and killing tests and cannot substitute for experimentally demonstrating an actionable collateral trade-off.

The learning tier runs in parallel: baseline and escape sequencing with selective assays updates receptor annotations, bank coverage, escape routes, and model ranges.

Figure 3 condenses this sequence into five prospective validation steps, three outcome branches, the Section 3.2 claim gates, and a cross-case learning tier, presented as a prospective study design for validation rather than as bedside treatment guidance.

**Figure 3:**
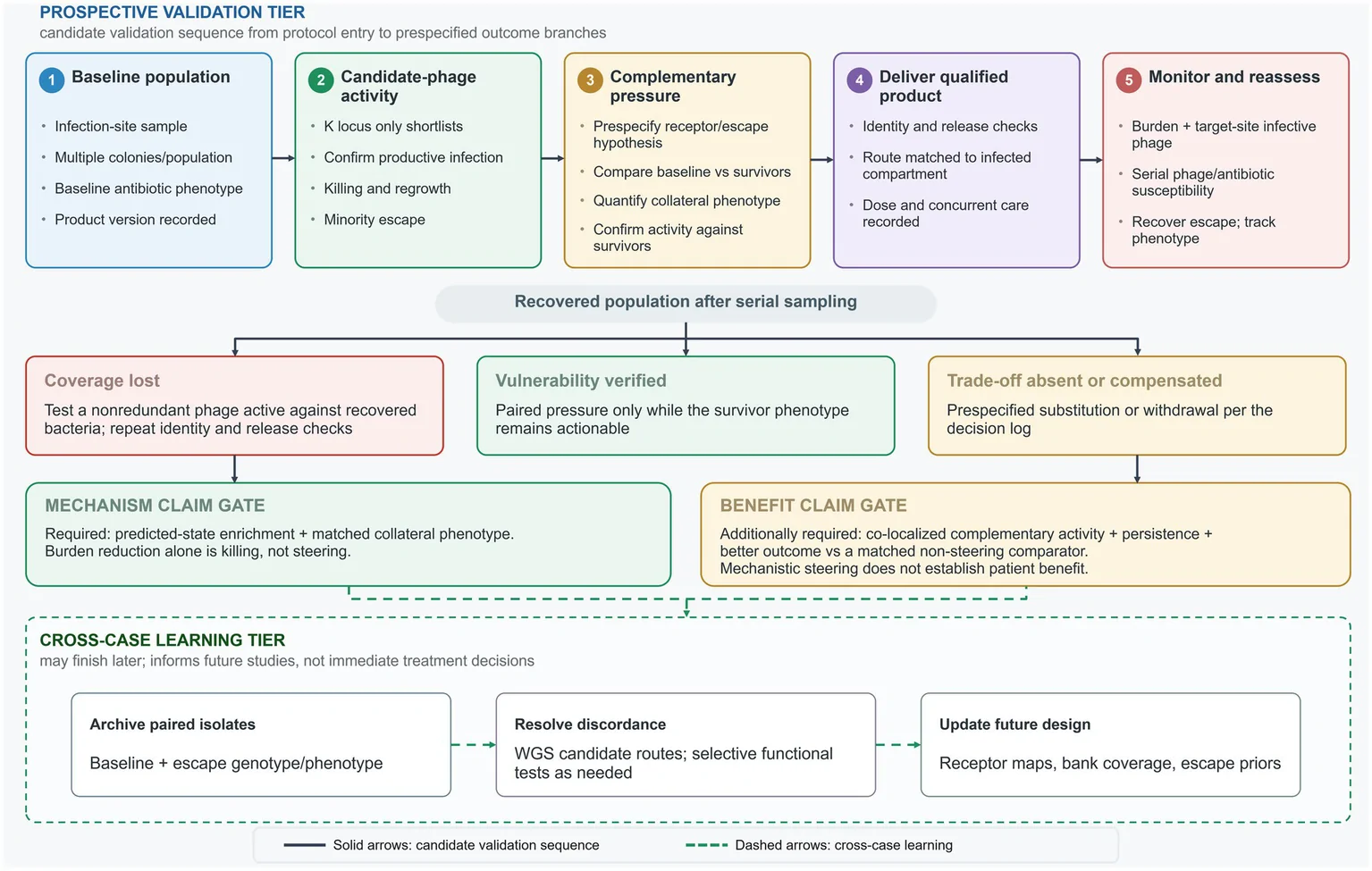
Simplified prospective validation framework for *Acinetobacter baumannii* evolutionary steering. This framework presents a prospective study design rather than a patient-management algorithm. Five prospective steps move from representative baseline sampling through candidate-phage activity testing, complementary-pressure validation, qualified-product delivery, and serial monitoring with prespecified outcome branches. The mechanism and benefit gates apply the criteria defined once in Section 3.2 for mechanistic steering and clinical benefit. Paired isolates and unresolved discordance feed a separate cross-case learning tier that informs future studies rather than immediate treatment decisions.

## 6 Clinical constraints on *Acinetobacter baumannii* evolutionary steering

This architecture is interpretable only if a qualified product is released within the treatment window, reaches the compartment, and links to longitudinal monitoring and prespecified adaptation; accordingly, the following subsections delineate pharmacological and evolutionary constraints that can invalidate steering, rather than prescribing recommendations for current patient management.

### 6.1 Reciprocal evolutionary adaptation and resistance dynamics

Under the Section 3.2 criteria, rapid resistance does not establish steering: in one decontamination system, resistance appeared within 24 h and an early 15–20% growth deficit was no longer significant at 24 h (Chen et al., 2025), establishing neither sustained compensation nor a durable therapeutic window. Serial sampling should distinguish regulation, alternative escape, clonal replacement, and compensation using burden, regrowth, genotype, susceptibilities, and phenotype; therapeutic substitution additionally requires a qualified product active against recovered bacteria.

Prospective trial protocols should evaluate continuation, rematching, and stopping criteria rather than inferring management endpoints from cumulative phage dose. Candidate failure states include lost productive coverage, uncorrectable dual resistance, compensation, toxicity, or exposure failure. These are validation variables to be applied within an approved study or other applicable clinical framework rather than as recommendations for current care.

### 6.2 *Acinetobacter baumannii*-specific product quality: LOS, OMVs, and host residues

Propagation creates a product-quality problem: crude lysates can contain LOS or pyrogen, cytotoxic OMVs, host DNA and protein, culture medium residues, non-infective particles, and induced prophages (Li et al., 2015); downstream purification can reduce infective yield, and manufacturing controls cannot eliminate LOS subsequently released *in vivo* (Szermer-Olearnik and Boratyński, 2015).

Manufacturing standards require qualified seed lots and propagation hosts, controlled passage and harvest cycles, validated impurity reduction, and aseptic formulation; product release must document identity, infective potency, microbiological quality, host residues, undesirable genomic cargo, stability, and batch consistency. Host or process changes require comparability and renewed release because impurity clearance and phage recovery are product-specific (European Directorate for the Quality of Medicines and HealthCare, 2024; European Medicines Agency, 2025).

### 6.3 Compartmental exposure as a condition for steering

Phage and companion pressure must overlap in the compartment while the phenotype remains actionable. Net amplification requires production to exceed inactivation and clearance; neither dose nor disappearance proves target-site activity (Tan et al., 2024; Delattre et al., 2022). In mice, vB_AbaSt_W16 showed route- and model-specific distribution and PEGylation-dependent circulation, findings that cannot be directly extrapolated across distinct phage formulations or patient populations (Choi et al., 2025, 2026). Prospective protocols should evaluate whether route, redosing, rematching, or withdrawal track target-site phage, burden, susceptibility, phenotype, neutralization, and toxicity; inadequate target-site exposure falsifies clinical exploitation within that study even if the predicted escape state was enriched.

### 6.4 Adaptive product changes, monitoring, and evidence attribution

A qualified bank and master protocol may permit rapid matching and prespecified substitution, but each product change requires documented coverage loss, renewed activity against recovered bacteria, release testing, and product-version traceability (Nagel et al., 2022); estimand, comparator, allocation, substitution, sampling, and stop criteria must be prespecified in one decision log. Access authorization does not establish quality: US expanded access remains investigational, European Pharmacopoeia chapter 5.31 provides informational quality guidance, and the EMA guideline remains in draft (U.S. Food and Drug Administration, 2026; European Directorate for the Quality of Medicines and HealthCare, 2024; European Medicines Agency, 2025), requiring jurisdiction-specific engagement, a prequalified bank, and prespecified comparability.

Feasibility should be measured by time to an active released product, failed matches, repeat sampling, manufacturing and release burden, hospital-resource use, recurrence, adverse events, survival, and access. No *Acinetobacter baumannii*-specific cost-effectiveness analysis was identified in the literature reviewed here. Economic evaluation should compare diagnostics, matching, banking, manufacture, release, transport, repeat sampling, failed matches, and waste with standard care, measuring resource use, recurrence, survival, and quality of life.

Ecological monitoring is retained only where it can delimit or invalidate the strategy: prospective studies must distinguish infective shedding from detected DNA sequences, quantify encapsidated host DNA and horizontal transfer risk, and evaluate environmental persistence and non-target microbiome perturbations. Microbiome editing requires strain-level targets, preserved non-target function, longitudinal outcomes, and transfer tests; containment follows the measured risk.

## 7 Conclusion

This review frames evolutionary steering in CRAB as a testable experimental hypothesis rather than an established therapeutic class effect. Across the evaluated literature, direct, matched phage-escape evidence remains narrowly concentrated in defined capsule-associated systems. In contrast, non-capsular envelope routes—such as outer-membrane proteins (OMPs) and lipooligosaccharide (LOS) determinants—currently represent indirect, candidate observations that lack complete paired steering verification. Furthermore, paradigms borrowed from other organisms (such as *Pseudomonas* OprM steering or the *Escherichia coli* TolC counterexample) serve strictly as cross-species mechanistic hypotheses rather than validated targets in *Acinetobacter baumannii*.

The criteria formalized in Section 3.2 separate mechanistic steering from clinical benefit, preventing passive bacterial killing, isolated phage resistance, or unmeasured protocol adaptations from being conflated with therapeutic steering. Generality remains conditional on genotype, strain, compartment, exposure, and compensation.

Priority evidence is paired and time-resolved: baseline and escape genotypes linked to quantitative phenotypes, target-site exposure, and prospective comparisons with predefined adaptation and clinical outcomes. While genomic sequencing clarifies escape trajectories, it cannot substitute for rapid phenotypic testing; the principal translational gaps remain narrow capsule-associated evidence, an absence of controlled comparisons, and limited compartmental exposure and durability data.
